# Perfluorooctanoic Acid for Shotgun Proteomics

**DOI:** 10.1371/journal.pone.0015332

**Published:** 2010-12-30

**Authors:** Chandra Sekhar Rao Kadiyala, Sara E. Tomechko, Masaru Miyagi

**Affiliations:** 1 Case Center for Proteomics and Bioinformatics, Case Western Reserve University, Cleveland, Ohio, United States of America; 2 Department of Pharmacology, Case Western Reserve University, Cleveland, Ohio, United States of America; 3 Department of Ophthalmology and Visual Sciences, Case Western Reserve University, Cleveland, Ohio, United States of America; University Paris Sud, France

## Abstract

Here, we describe the novel use of a volatile surfactant, perfluorooctanoic acid (PFOA), for shotgun proteomics. PFOA was found to solubilize membrane proteins as effectively as sodium dodecyl sulfate (SDS). PFOA concentrations up to 0.5% (w/v) did not significantly inhibit trypsin activity. The unique features of PFOA allowed us to develop a single-tube shotgun proteomics method that used all volatile chemicals that could easily be removed by evaporation prior to mass spectrometry analysis. The experimental procedures involved: 1) extraction of proteins in 2% PFOA; 2) reduction of cystine residues with triethyl phosphine and their *S*-alkylation with iodoethanol; 3) trypsin digestion of proteins in 0.5% PFOA; 4) removal of PFOA by evaporation; and 5) LC-MS/MS analysis of the resulting peptides. The general applicability of the method was demonstrated with the membrane preparation of photoreceptor outer segments. We identified 75 proteins from 1 µg of the tryptic peptides in a single, 1-hour, LC-MS/MS run. About 67% of the proteins identified were classified as membrane proteins. We also demonstrate that a proteolytic ^18^O labeling procedure can be incorporated after the PFOA removal step for quantitative proteomic experiments. The present method does not require sample clean-up devices such as solid-phase extractions and membrane filters, so no proteins/peptides are lost in any experimental steps. Thus, this single-tube shotgun proteomics method overcomes the major drawbacks of surfactant use in proteomic experiments.

## Introduction

Shotgun proteomics experiment uses surfactants in a major role to achieve efficient extraction and digestion of proteins. The surfactants widely used in shotgun proteomics can be classified as ionic (e.g., SDS), nonionic (e.g., Triton-X), and acid cleavable surfactants (e.g., RapiGest). In brief, a typical strategy for shotgun proteomics of samples containing membrane proteins begins with protein extraction from cells or tissues in the presence of a surfactant, then cystine residues are reduced and alkylated under denaturing conditions, and then the surfactant is subsequently removed by acetone precipitation [1] or by exchange with urea on a standard filtration device [2]. The resulting acetone precipitate is generally solubilized either in a strong chaotropic agent such as urea or guanidine hydrochloride (Gdn-HCl) or in a surfactant, and then subjected to proteolytic digestion. After the digestion, the chaotropic agent are removed by a reverse-phase solid phase extraction. However, when a surfactant was used, it cannot be removed easily from the digest. Because surfactants are hydrophobic in nature, they cause peak broadening and suppress the ionization of peptides in the subsequent LC-MS/MS analysis [3]. Thus, the surfactants used must be removed prior to LC-MS/MS analysis. Many research groups have described removal of surfactants from peptide mixtures either by ion exchange chromatography [3], by phase transfer [4], or by washing with chlorinated solvents while peptides are captured on a reversed phase cartridge [5]. These extra preparation steps have the major drawback of losing peptides to the stationary phase during the procedures, which cannot be afforded when sample amount is limited. To avoid this problem, acid labile surfactants have been developed that can be cleaved between the hydrophobic and hydrophilic part of the surfactants after the protein digestion [6], [7]. The hydrophobic part precipitates upon the acid cleavage, allowing its removal from the digest. The hydrophilic part does not interfere with the subsequent LC-MS/MS analysis. However, it has been reported that hydrophobic peptides are lost from the digest due to their interactions with the precipitated hydrophobic part of the surfactant [8]. Thus, a method is needed that does not lead to the loss of peptides.

Our laboratory has been in search of an ideal surfactant that can effectively solubilize hydrophobic proteins, is compatible with proteases, and can easily be removed from the samples prior to mass spectrometry analysis. We predicted that surfactants that have low boiling points have potential to meet these needs. We tested two volatile surfactants, pentafluorooctanoic acid (PFOA, CAS Registry No.: 335-67-1) and N,N-dihexylamine (CAS Registry No.: 143-16-8) whose boiling points are 188.0 and 193.5°C at 760 Torr, respectively. Since PFOA was superb at efficiently solubilizing proteins compared to dihexylamine, we focused on PFOA. PFOA is a synthetic, stable perfluorinated C8 carboxylic acid that is generally used in preparation of fluoropolymers, which are used in the manufacture of a wide variety of products such as nonstick surfaces on cookware (Teflon) and protective finishes on carpets and clothing. Other applications include aerospace, automotive, chemical processing, semiconductor manufacturing, information, and telecommunication [9]. Fluorinated surfactants have also been used by various laboratories to solubilize membrane proteins [10]. In mass spectrometry applications, PFOA has been used as an ion-pair agent in LC-MS analysis [11], and as a matrix additive in MALDI-MS to enhance ionization of lipoproteins [12]. To date, however, the use of PFOA for proteomic applications has not been reported.

Here, we show that PFOA efficiently solubilizes membrane proteins and is compatible with trypsin. By utilizing this volatile surfactant and adapting the method by Hale and co-workers for the reduction and *S*-alkylation of cystine residues using volatile reagents [13], we developed a single-tube shotgun proteomics method. The detailed experimental workflow is described and the applicability of the method is demonstrated by analyzing a membrane preparation from photoreceptor outer segments.

## Materials and Methods

### Materials

PFOA was purchased from TCI America (Portland, OR). Oxygen-18 enriched water was obtained from Isotec (Miamisburg, OH). Sequencing–grade, modified, porcine trypsin was purchased from Promega (Madison, WI). All other chemicals and materials were either reagent grade or of the highest quality commercially available.

### Preparation of photoreceptor outer segments membrane pellet

Bovine retinas obtained from WL Lawson Company (Omaha, NE) were used to prepare the photoreceptor outer segment (OS) by sucrose density ultracentrifugation [1]. All solvents used for bovine OS preparations contained protease inhibitors (1 mM EDTA, 0.2 mM PMSF, 0.7 µg/ µL leupeptin, and 0.5 µg/ µL pepstatin A) to inhibit protein degradation and 100 µM diethylenetriamine pentaacetic acid (DTPA) to inhibit oxidation. After the OS were isolated, the purified OS solution (10 µL) was mixed with 100 µL of 100 mM ammonium bicarbonate (ABC) that contained the protease inhibitors, centrifuged at 15,000 *g* for 10 minutes, and then the supernatant was discarded. The precipitated OS membrane pellet was washed twice with 100 µL of 100 mM ABC and used for the proteomic analysis described below.

### Protein solubilization efficiencies of various solubilizing agents

The protein solubilization efficiencies of different solubilizing agents were studied by solubilizing the OS membrane protein pellet in 50 µL of 100 mM ABC containing either 1% SDS (w/v), 1% PFOA (w/v), 4 M urea, or 4 M guanidine-HCl (Gdn-HCl). The solubilized pellet solution was sonicated with a VirSonic 100 ultrasonic cell disrupter (SP Scientific, Gardiner, NY) three times at 4.5 kHz of ultrasonic frequency for 9 seconds with 3-minute intervals between the sonications. The resulting protein extract was centrifuged at 15,000 *g* for 10 minutes, and the solubilized proteins in the supernatant were quantified using a DC protein assay kit (Bio-Rad, Hercules, CA).

### Effect of PFOA on trypsin activity

To measure the amidase activity of 100 nM trypsin, we tracked the hydrolysis of 2 mM Ac-Lys-*p-*nitro aniline hydrochloride through absorbance increase at 405 nm over 3 min in 100 mM ABC using an ELISA plate reader (Thermo-Fisher Scientific, Waltham, MA). The enzyme activity observed in the absence of solubilizing agents was the control and was considered 100%. The activity in the presence of different concentrations of surfactant (SDS or PFOA) or chaotropic agents (urea or Gdn-HCl) was expressed with respect to the control under the same experimental conditions.

### Single-tube proteolytic ^18^O labeling

**Figure 1:**
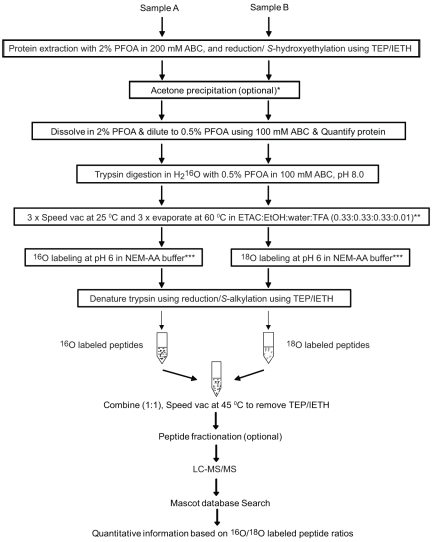
The proteolytic ^18^O labeling procedure uses a single tube. *The photoreceptor OS membrane protein was prepared by precipitation with acetone to remove the lipids from the plasma and disc membranes, but depending on the nature of sample, this step may not be required. If acetone precipitation is not required, the excess of the TEP/IETH can be removed by speed-vac at 45°C. ** After every reconstitution step the sample was sonicated in a water bath for 10 sec. Formic acid can also be used instead of TFA. ***100 mM ammonium formate buffer (pH 6) can also be used. TEP (triethylphosphene), IETH (iodoethanol), ABC (ammonium bicarbonate), PFOA (perfluorooctanoic acid), ETAC (ethylacetate), EtOH (ethanol), TFA (trifluoroacetic acid), and NEM-AA (n-ethyl morpholine-acetic acid).

**Note:** TEP and IETH stock solutions were prepared in pure acetonitrile. All operations with PFOA removal by evaporation under atmospheric pressure needs to be performed in fume hood for safety reasons. NEM-AA buffer in H_2_^18^O was prepared by mixing 491 µL H_2_^18^O, 2.95 µL glacial acetic acid and 6 µL of NEM. The pH of this solution becomes around 6. When greater than 200 µg of protein samples are processed, we recommend to use a larger sample tube and larger volume of the reconstitution solution (ethanol∶ethylacetate∶water∶TFA).

The entire experimental workflow of a single-tube proteolytic ^18^O labeling is shown in Figure 1. In this hypothetical proteomic experiment, two identical OS membrane pellets from 10 µL of OS solution in 1.5-mL low retention tubes (Fisher Scientific, Pittsburgh, PA) were dissolved in 50 µL of 2% PFOA in 200 mM ABC by applying ultrasound energy at 4.5 kHz three times for 9 seconds with a 3-minute pause between the strokes. The extracted OS membrane proteins were reduced with 21 mM triethyl phosphine (TEP) at 45°C for 1 hr and then *S*-alkylated by 58 mM iodoethanol (IETH) at 45°C for 2 hr in the dark. Then, the proteins were precipitated by mixing with a 6-fold excess volume of ice-cold acetone and incubated 2 hr at −20°C. The acetone precipitation removes lipids from the protein sample [1]. The precipitated proteins were then centrifuged at 2400 *g* for 10 minutes in a table-top centrifuge, and the pellet was washed twice with ice-cold acetone. The protein pellet was air dried for 10 minutes, and then redissolved in 50 µL of 2% PFOA in 200 mM ABC by sonication in a water bath for 10 min in a Bransonic Ultrasonic 2510R-MT (Danbury, CT). The protein solution was then diluted in 100 mM ABC to 0.5% PFOA, and the amount of dissolved protein was determined by the DC protein assay kit (Bio-Rad, Hercules, CA). A total of 25 µg protein in 200 µL of 0.5% PFOA in both the tubes was digested in H_2_^16^O by trypsin (1∶100 substrate to protein ratio w/w) at 37°C for 18 h. Following the digestion, the digest was dried in a speed-vac concentrator (Thermo-Fisher Scientific Model SPD121P-120) at 25°C under low pressure of <10 mTorr. The dried digest was subjected to three cycles of reconstitution in 100 µL of ethanol∶ethylacetate∶water∶TFA (0.33∶0.33∶0.33∶0.01, v/v/v/v) and evaporation in the speed-vac concentrator at 25°C under low pressure of <10 mTorr, followed by another three cycles of reconstitution in 100 µL of ethanol∶ethylacetate∶water∶TFA (0.33∶0.33∶0.33∶0.01, v/v/v/v) and evaporation in a speed-vac concentrator at 60°C under atmospheric pressure (without applying vacuum). It should be noted that the concentrator rotor still needs to be rotated during the evaporation process at the atmospheric pressure to minimize adsorption of the peptides to the tube. After every reconstitution step the sample was sonicated in a water bath for 10 sec. The 100 µL solution was completely dried typically in 60 min. The six cycles of solubilization and evaporation procedure was needed to thoroughly remove the PFOA. Next, the peptides from each tube were dissolved in 25 µL of 100 mM N-ethylmorpholine-acetic acid (NEM-AA) buffer at pH 6 that was made with H_2_^16^O or H_2_^18^O, respectively. The peptides were then incubated with trypsin (1∶50 trypsin to peptide ratio w/w) at 25°C for 18 hr to incorporate ^16^O and ^18^O, respectively, into the carboxyl termini of the peptides. After the labeling, 75 µL of pure isopropyl alcohol was added to denature the trypsin, and the solutions were adjusted to approximately pH 8 by adding 1 M ABC dissolved either in H_2_^16^O or H_2_^18^O. The trypsin was then inactivated completely by reduction with 21 mM TEP at 45°C for 1 hr followed by *S*-alkylation by 58 mM IETH at 45°C for 2 hr in the dark. The resulting ^16^O and ^18^O labeled peptides were mixed in a 1∶1 ratio and all the volatile reagents were then removed in a Speed-vac concentrator at 45°C, and 1 µg of the mix was analyzed by LC-MS/MS.

### LC-MS/MS analysis

LC-MS/MS analyses used a UltiMate 3000 LC systems (Dionex Inc., San Francisco, CA) interfaced to a LTQ-Orbitrap XL mass spectrometer (Thermo-Finnigan, Bremen, Germany). The platform was operated in the nano-LC mode using the standard nano-ESI API source fitted with a PicoTip emitter that had an uncoated fitting and 10-µm spray orifice (New Objective, Inc., Woburn, MA). The solvent flow rate through the column was maintained at 300 nL/min using a 1∶1000 splitter system. The protein digest (typically 5 µL) was injected into a reversed-phase C18 PepMap trapping column of 0.3×5 mm with a 5-µm particle size (Dionex Inc.) equilibrated with 0.1% formic acid/2% acetonitrile (v/v). It was washed for 5 min with the equilibration solution at a flow rate of 25 µL/min by using an isocratic loading pump operated through an auto sampler. Next, the trapping column was switched in-line with a reversed-phase C18 Acclaim PepMap 100 column of 0.075×150 mm (Dionex Inc.) and the peptides were chromatographed using a linear gradient of acetonitrile from 6% to 50% in aqueous 0.1% formic acid over 50 minutes at the 25 µL/min flow rate. The eluate was directly introduced to the mass spectrometer. The mass spectrometer was operated in a data-dependent MS to MS/MS switching mode, with the five most intense ions in each MS scan subjected to MS/MS analysis. The full MS scan was performed at a resolution of 60,000 in the Orbitrap detector and the MS/MS scans were performed in the ion trap detector in collision-induced dissociation (CID) mode. The threshold intensity for the MS/MS trigger was always set at 1000. The fragmentation was carried out using the CID mode with a normalized collision energy of 35. The data was entirely collected in the profile mode for the full MS scan and the centroid mode for the MS/MS scans. The dynamic exclusion function for previously selected precursor ions applied the following parameters: repeat count of 2, repeat duration of 45 seconds, exclusion duration of 60 seconds, and exclusion size list of 150. Xcalibur software (version 2.0.7, Thermo-Finnigan Inc., San Jose, CA) was used for instrument control, data acquisition, and data processing.

### Protein identification

Proteins were identified by comparing all of the experimental peptide MS/MS spectra to the Swiss-Prot (version 57) database using Mascot database search software (version 2.1.04, Matrix Science, London, UK). The *S*-hydroxyethylation of cysteine was set as a fixed modification while the oxidation of methionine to methionine sulfoxide and the modification of C-terminal with ^18^O were variable modifications. The mass tolerance was set to 10 ppm for the precursor ion and to 0.8 Da for the product ion. Strict trypsin specificity was applied, allowing for two missed cleavages. Only peptides with a minimum score of 20 were considered significant. Scaffold software (Version Scaffold-2_06_00, Proteome Software, Inc., Portland, OR) was used to validate MS/MS-based peptide and protein identification. Peptide identifications were accepted if they could be established at an ion score greater than 20 as specified by the Peptide Prophet algorithm [14]. Protein identifications were accepted if they could be established at greater than 95% probability and contained at least two identified peptides. Protein probabilities were assigned by the Protein Prophet algorithm.

### Calculation of ^16^O/^18^O-Peptide Ratio

In-house software (Relative Quantification O18.2.2.2) employing a least-squares regression algorithm [15] was used for the calculation of ^16^O/^18^O peptide ratios. This software plots ^16^O/^18^O-peptide intensities of all peptides identified from the same protein, and the slope of the linear regression fit is used as a ^16^O/^18^O peptide ratio for that protein. Only proteins with *R* ^2^≥0.85 and a linear regression *F*-probability greater than 0.85 are reported as quantified proteins. Proteins with *R* ^2^ values or *F*-probabilities out of our range were manually investigated for possible peptide outliers. An obvious outlier was defined as a peptide whose removal changed the protein *R* ^2^ value by more than 0.2 or increased the *F*-probability to >0.85. If an obvious outlier was detected, it was removed from the peptide list. The slope of the linear regression fit was obtained from the plot of ^18^O intensity on the y-axis vs. the ^16^O intensity of the same peptide on x-axis. The slope value normalized the individual peptide ratios. This is expected to decrease the influence of experimental error (e.g., pipetting error during sample mixing) on the calculated ratios.

## Results and Discussion

### Protein solubilization efficiency of PFOA

The total protein amounts solubilized from the OS membrane protein pellet by various solubilizing agents are shown in Table 1. We found that 100 mM ABC could solubilize 9.2 µg of protein, 1% PFOA could solubilize 47.4 µg protein, and 1% SDS could solubilize 55.1 µg protein. So, PFOA and SDS solubilized over 5-fold more protein than 100 mM ABC. Surprisingly, Gdn-HCl solubilized only 7.3 µg and urea only 8.1 µg. These results demonstrate that PFOA can solubilize protein at an efficiency comparable to SDS, and considerably higher than urea and Gdn-HCl. The results also suggest that urea and Gdn-HCl may not help in solubilizing membrane proteins in proteomic applications. Because over 80% of the total weight of OS membrane protein is the seven-transmembrane receptor rhodopsin [16], the results should be considered most relevant for highly hydrophobic integral membrane proteins.

**Table 1:** Solubilization of membrane proteins by different reagents.

Solubilization agent	Protein amount solubilized
	(µg/10 µL OS membrane preparation)
100 mM ABC	9.2±2.0
25 mM SDS (0.8% w/v)	55.1±6.7
25 mM PFOA (1% w/v)	47.4±4.4
4 M Urea	8.1±3.4
4 M Gdn-HCl	7.3±4.1

Data are means ± standard deviation from triplicate experiments.

### Effect of PFOA on the trypsin activity

**Figure 2:**
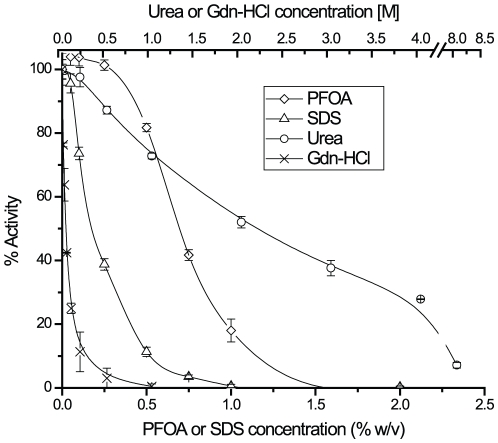
Different solubilizing agents affected the activity of trypsin. The amidase activity of trypsin was measured by monitoring the initial rate of the hydrolysis of Ac-Lys-*p*-nitroanilide. Each line in the graph represents the concentration dependent decrease in tryptic activity in the presence of various concentrations of PFOA (◊), SDS (Δ), urea (○), and Gdn-HCl (x).

We tested the activities of porcine trypsin in the presence of various concentrations of PFOA and compared the activity to other protein denaturation agents (Figure 2). Trypsin retained full activity in PFOA concentrations up to 0.25%, and more than 80% of its activity in 0.5% PFOA. In contrast, trypsin retained only about 10% activity at 0.5% SDS. The results suggest that concentrations of PFOA below 0.5% can be used for protein digestion without drastically inhibiting the tryptic activity. The effect of urea and Gdn-HCl were also compared (Figure 2). Trypsin retained approximately 50% of its activity in 2 M urea and 20% in 4 M urea, while it retained only 40% in 0.1 M Gdn-HCl and 20% in 0.25 M Gdn-HCl. These results were comparable to our previous report on porcine trypsin [17].

### Removal of PFOA from peptide mixture

The major hurdle in the development of this method turned out to be the removal of PFOA from the peptide mixture after digestion. We initially tried removing PFOA from a tryptic digest of OS membrane proteins dissolved in 0.1% formic acid and 60% acetonitrile, which is a commonly used solvent mixture in proteomic applications. However, we were unsuccessful even after repeated evaporations in a speed-vac concentrator under low pressure of <10 mTorr. The peptide peaks broadened in LC-MS/MS (data not shown), suggesting that a considerable amount of PFOA remained with the peptide sample. We believe that the remaining PFOA molecules in the digest are mainly the ones interacting with peptides through ionic and/or hydrophobic interactions.

To remove PFOA completely from the peptide samples, we tested several factors including solvents, temperatures, and vacuum conditions that are likely to affect the evaporation process of PFOA. The solvents water, methanol, ethanol, ethyl acetate, acetonitrile, n-propanol, dimethyl formamide, and dimethylsulfoxide were tested individually and in mixes of various combinations and ratios. The temperatures of 30, 40, 50, and 60°C and the pressures of 1 mTorr and 760 Torr were tested. A small quantity of TFA (1%) was included in all the solutions to protonate the carboxyl group of PFOA (the p*K*_a_ value of PFOA is 3.8 at infinite dilution [18]), which is expected to disrupt the ionic interactions between PFOA and peptides, therefore helps evaporating PFOA. A total of 50 µg of tryptic digest of bovine serum albumin (BSA) was dissolved in 100 µL of 0.1% v/v PFOA in the various solvents with 1% TFA. The PFOA was evaporated at the different temperatures and pressures. After the evaporation, the resulting BSA digest was redissolved in 0.1% formic acid and 50% acetonitrile, and analyzed by flow injection MS. The residual PFOA was measured by monitoring the extent of (M–H)^−^ ion of PFOA (m/z 413). We found that the PFOA amount in the BSA digest was decreased below detectable level after three cycles of reconstitution in 100 µL of ethanol∶ethylacetate∶water∶TFA (0.33∶0.33∶0.33∶0.01, v/v/v/v) and evaporation at 60°C under atmospheric pressure of 760 Torr (data not shown).

**Figure 3:**
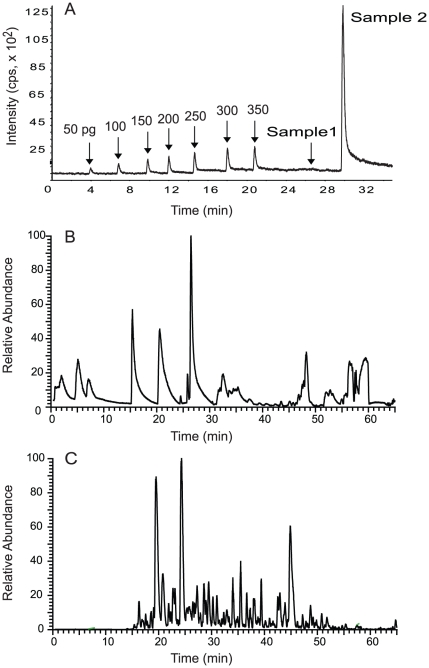
Total ion current chromatograms of the tryptic digest of OS membrane proteins. (A) Residual PFOA in the tryptic digest of OS membrane proteins quantified by flow injection analysis. Different concentrations of the PFOA standard solution (50–350 pg) and samples in 1 µL of 0.1% formic acid and 50% acetonitrile were injected into a flowing carrier stream consisting of 0.1% formic acid and 50% acetonitrile at 40 µL/min that was directly connected to a mass spectrometer (QStar, Applied Biosystems, Foster City, CA) equipped with an electrospray ion source. The (M–H)^−^ ion (m/z 413) of PFOA was monitored. An aliquot of the digest was analyzed by flow injection analysis after three cycles of evaporation in ethanol∶ethylacetate∶water∶TFA (0.33∶0.33∶0.33∶0.01, v/v/v/v) at 25°C in a speed-vac concentrator under low pressure of <10 mTorr (Sample 2). The remaining digest was subjected to another three cycles of evaporation in ethanol∶ethylacetate∶water∶TFA (0.33∶0.33∶0.33∶0.01, v/v/v/v) at 60°C in a speed-vac concentrator under atmospheric pressure of 760 Torr (Sample 1). (B) 1 µg of the digest from sample 2 analyzed by LC-MS/MS. (C) 1 µg of the digest from sample 1 analyzed by LC-MS/MS.

The digest of OS membrane protein (25 µg) in 200 µL of 0.5% PFOA in 100 mM ABC (total PFOA amount  = 1 mg) was subjected to speed-vac, and then three cycles of reconstitution in 100 µL of ethanol∶ethylacetate∶water∶TFA (0.33∶0.33∶0.33∶0.01, v/v/v/v) and evaporation in a speed-vac concentrator under low pressure of <10 mTorr. An aliquot (2.5 µg) was redissolved in 50 µL of 0.1% formic acid and 50% acetonitrile, and 1 µL of which was analyzed by flow injection MS (Figure 3A, Sample 2). The residual PFOA was estimated to be 12 µg, which corresponds to 1.2% of the initial amount. When 1 µg of the same digest was analyzed by LC-MS/MS, the peptide peak widths were broader than normal (Figure 3B), suggesting that a small amount of PFOA remained in the digest can interfere with the chromatography. In order to remove the residual PFOA completely, the rest of the digest (22.5 µg) was subjected to another three cycles of reconstitution in 100 µL of ethanol∶ethylacetate∶water∶TFA (0.33∶0.33∶0.33∶0.01, v/v/v/v) and evaporation at 60°C under atmospheric pressure of 760 Torr. An aliquot (2.5 µg) was redissolved in 50 µL of 0.1% formic acid and 50% acetonitrile, and 1 µL of which was analyzed by flow injection MS (Figure 3A, Sample 1). As can be evident in the figure, PFOA was not detectable, suggesting the virtually complete removal of PFOA from the digest.

The PFOA free tryptic digest of OS membrane proteins (1 µg) was analyzed by LC-MS/MS (Figure 3C). The peak broadening problem caused by PFOA was obviously fixed after the removal of PFOA. A total of 75 proteins were identified by at least two unique peptides from the single 1 hr LC-MS/MS run of the tryptic digest of OS membrane proteins, and 67% of these, or 50 proteins, were classified as membrane proteins (Table S1). This result demonstrates the usefulness of PFOA for identifying membrane proteins in shotgun proteomics.

### Single tube proteolytic ^18^O labeling method

**Figure 4:**
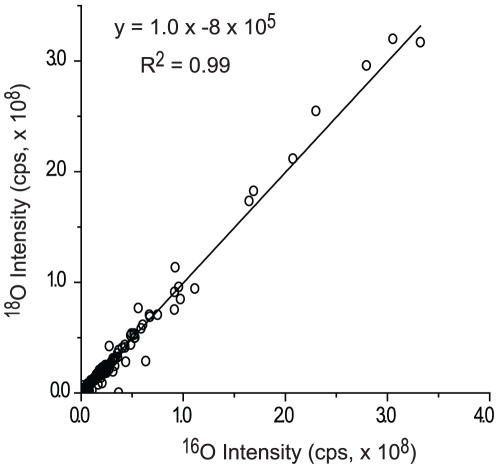
Plots compare the intensities of ^16^O- and ^18^O-labeled peptides. Linear regression analysis was performed on a total of 377 peptides. The equations and R^2^ values for the regression line are shown.

Proteolytic ^18^O labeling is one of the most widely used quantitative shotgun proteomics methods, and it determines the relative ratios of individual proteins between two samples [19], [20], [21]. In this method, protein digestion and ^18^O labeling, which are catalyzed by the same protease, can be decoupled [22]. In a typical decoupled experiment, proteins are digested before being subjected to ^18^O labeling. Therefore, we attempted to incorporate the ^18^O labeling procedures into the protocol. The PFOA-free tryptic digests of the OS membrane proteins were reconstituted in NEM-AA buffer pH 6 made with either H_2_^16^O or H_2_^18^O (Figure 1). The peptides were then incubated with trypsin to incorporate ^16^O or ^18^O into the carboxyl termini of the peptides. After the labeling, the trypsin was inactivated by using volatile reagents to reduce and alkylate its cystine residues. The ^16^O and ^18^O labeled peptide samples were mixed in 1∶1 ratio and the excess reagents were removed in a speed-vac concentrator. When 1 µg of the mixed peptide sample was analyzed by LC-MS/MS, we quantified about 377 peptides. Figure 4 plots the ^16^O- versus ^18^O-labeled peptide intensities observed in the LC-MS/MS and shows the regression line (R^2^ = 0.99) from linear regression analyses. This result demonstrates that ^18^O labeling can be successfully performed in a single tube. This single-tube, quantitative, shotgun proteomics method does not require proteomic samples to be transferred out of the original reaction tube until the ^18^O labeling is completed, which limits the loss of samples only to the tube used and thus assures high recovery of the peptides from minute quantities of tissue samples.

### Advantages and drawbacks of the single-tube proteolytic ^18^O labeling method

The highly efficient protein solubilization of our PFOA method is comparable to SDS, the gold standard surfactant for protein solubilization. The volatile nature of all the solvents, reagents, and buffers used in the method allows them to be removed by evaporation. This evaporation means we expect no loss of proteins or peptides prior to mass spectrometry analysis except loss of the peptides due to the adsorption on the tube used during the sample preparation [23], assuming that no proteins or peptides are volatile unless derivatized [24]. This method would be especially valuable when available sample amounts are limited. After PFOA was removed from protein digests, we have successfully fractionated peptides by strong cation exchange chromatography and by alkaline-pH reverse-phase chromatography (unpublished results), therefore such peptide fractionation methods can be incorporated into the method. The lengthy evaporation process to remove the PFOA is not a major setback, if the sample is in short supply. This evaporation process could be accelerated by finding a better solvent(s) from which PFOA can be efficiently evaporated at a low pressure of <10 mHg, which is the typical operation pressure of a speed-vac concentrator. Our laboratory is putting continuous effort to come up with quicker way of PFOA removal from protein digests.

## Supporting Information

Table S1 **OS proteins identified.**
(XLS)

## References

[1] Hajkova D, Imanishi Y, Palamalai V, Rao KC, Yuan C (2010). Proteomic Changes in the Photoreceptor Outer Segment upon Intense Light Exposure.. J Proteome Res.

[2] Wisniewski JR, Zougman A, Nagaraj N, Mann M (2009). Universal sample preparation method for proteome analysis.. Nat Methods.

[3] Vissers JP, Hulst WP, Chervet JP, Snijders HM, Cramers CA (1996). Automated on-line ionic detergent removal from minute protein/peptide samples prior to liquid chromatography-electrospray mass spectrometry.. J Chromatogr B Biomed Appl.

[4] Masuda T, Tomita M, Ishihama Y (2008). Phase transfer surfactant-aided trypsin digestion for membrane proteome analysis.. J Proteome Res.

[5] Rey M, Mrazek H, Pompach P, Novak P, Pelosi L (2010). Effective removal of nonionic detergents in protein mass spectrometry, hydrogen/deuterium exchange, and proteomics.. Anal Chem.

[6] Norris JL, Porter NA, Caprioli RM (2003). Mass spectrometry of intracellular and membrane proteins using cleavable detergents.. Anal Chem.

[7] Yu YQ, Gilar M, Lee PJ, Bouvier ES, Gebler JC (2003). Enzyme-friendly, mass spectrometry-compatible surfactant for in-solution enzymatic digestion of proteins.. Anal Chem.

[8] Yu YQ, Gilar M, Gebler JC (2004). A complete peptide mapping of membrane proteins: a novel surfactant aiding the enzymatic digestion of bacteriorhodopsin.. Rapid Commun Mass Spectrom.

[9] Negri S, Maestri L, Esabon G, Ferrari M, Zadra P (2008). Characteristics, use and toxicity of fluorochemicals: review of the literature.. G Ital Med Lav Ergon.

[10] Shepherd FH, Holzenburg A (1995). The potential of fluorinated surfactants in membrane biochemistry.. Anal Biochem.

[11] Ishihama Y, Katayama H, Asakawa N (2000). Surfactants usable for electrospray ionization mass spectrometry.. Anal Biochem.

[12] Loo RR, Loo JA (2007). Matrix-assisted laser desorption/ionization-mass spectrometry of hydrophobic proteins in mixtures using formic acid, perfluorooctanoic acid, and sorbitol.. Anal Chem.

[13] Hale JE, Butler JP, Gelfanova V, You JS, Knierman MD (2004). A simplified procedure for the reduction and alkylation of cysteine residues in proteins prior to proteolytic digestion and mass spectral analysis.. Anal Biochem.

[14] Keller A, Nesvizhskii AI, Kolker E, Aebersold R (2002). Empirical statistical model to estimate the accuracy of peptide identifications made by MS/MS and database search.. Anal Chem.

[15] Eckel-Passow JE, Oberg AL, Therneau TM, Mason CJ, Mahoney DW (2006). Regression analysis for comparing protein samples with 16O/18O stable-isotope labeled mass spectrometry.. Bioinformatics.

[16] Papermaster DS, Dreyer WJ (1974). Rhodopsin content in the outer segment membranes of bovine and frog retinal rods.. Biochemistry.

[17] Kiser JZ, Post M, Wang B, Miyagi M (2009). Streptomyces erythraeus trypsin for proteomics applications.. J Proteome Res.

[18] Burns DC, Ellis DA, Li H, McMurdo CJ, Webster E (2008). Experimental pKa determination for perfluorooctanoic acid (PFOA) and the potential impact of pKa concentration dependence on laboratory-measured partitioning phenomena and environmental modeling.. Environ Sci Technol.

[19] Miyagi M, Rao KC (2007). Proteolytic 18O-labeling strategies for quantitative proteomics.. Mass Spectrom Rev.

[20] Fenselau C, Yao X (2009). 18O2-labeling in quantitative proteomic strategies: a status report.. J Proteome Res.

[21] Capelo JL, Carreira RJ, Fernandes L, Lodeiro C, Santos HM (2010). Latest developments in sample treatment for 18O-isotopic labeling for proteomics mass spectrometry-based approaches: a critical review.. Talanta.

[22] Yao X, Afonso C, Fenselau C (2003). Dissection of proteolytic 18O labeling: endoprotease-catalyzed 16O-to-18O exchange of truncated peptide substrates.. J Proteome Res.

[23] Pezeshki A, Vergote V, Van Dorpe S, Baert B, Burvenich C (2009). Adsorption of peptides at the sample drying step: influence of solvent evaporation technique, vial material and solution additive.. J Pharm Biomed Anal.

[24] Zaikin VG, Halket JM (2005). Review: derivatization in mass spectrometry-6. Formation of mixed derivatives of polyfunctional compounds.. Eur J Mass Spectrom (Chichester, Eng).

